# Intensive, Repeated Self-Report Measures: Should We Be Concerned About Changes in Data Quality Over Time?

**DOI:** 10.2196/68735

**Published:** 2026-01-14

**Authors:** Arthur A Stone, Stefan Schneider, Meynard J Toledo, Raymond Hernandez

**Affiliations:** 1 Department of Psychology University of Southern California Los Angeles, CA United States

**Keywords:** ecological momentary assessment, EMA, repeated measurements, invariance, self-report

## Abstract

Intensive, repeated self-report measures are an important tool for behavioral and medical researchers and practitioners who are concerned with the dynamic interplay among variables at a granular level. Many mobile health applications rely on accurate measurement of immediate states and environments for both assessment and intervention delivery. Techniques for capturing repeated momentary assessments yield data with several salutary qualities: recall bias is minimized relative to assessments that rely on much longer recall periods; measurements are taken in individuals’ everyday environments; and dense, repeated measures allow a new window into the processes transpiring between individuals and their environments. In this paper, we highlight several features of repeatedly completing momentary assessments that may change the nature or quality of the data collected over time. Several lines of inquiry are discussed that call into question the presumption that there is invariance in how people complete repeated assessments over time. A result of this possibility could be a reduction in data quality. We present 4 phenomena, with selected results, that may induce noninvariance in repeated measures: the amount of time required to complete assessments, the rate of missing data, the degree of careless responding, and the presence of several components of reactivity. In each of these areas, we found evidence that changes could occur over time, and we consider how data might be affected by such changes. Our conclusion is that researchers should be aware that changes can occur over time and that these changes may affect data quality.

## Introduction

Repeated self-report measures are central to intensive longitudinal methods that use momentary assessments, including ecological momentary assessment (EMA) [1,2], the experience sampling method [3], and ambulatory assessment methods [4]. Methodological strategies derived from momentary assessment studies form the backbone of optimal mobile health (mHealth) data acquisition for participant monitoring, in-the-moment treatment delivery (eg, just-in-time adaptive interventions), and real-time feedback on behavior and thoughts. As such, there is considerable value in recognizing the strengths and the potential limitations of these methods.

The advantages of repeated measures over static measures are numerous: they provide the opportunity to track trajectories of outcomes over time, enable exploration of lagged associations that may confer near-causal status, and reduce problems associated with retrospection [2,5-7]. Repeated-measures study designs are also part and parcel of many mHealth practices. Moreover, constructs often measured using momentary methods are pertinent for mHealth researchers; they include internal states (eg, pain intensity and quality, emotions, fatigue, perceived stress, symptoms, and cognitive status) and external (often observable) behaviors (eg, daily activities, social engagement, consumption, substance use, exogenous events, and location). Real-time self-reports may also be linked to ambulatory measures of physiological function (eg, heart rate, blood pressure, cortisol levels, and blood glucose) and to data routinely collected by smartphones (eg, location, step counts, and time taken to respond to questions) to provide insights into the dynamics of these variables. Finally, intensive, repeated measures are indispensable tools for monitoring populations, evaluating treatments in medical research, analyzing economic patterns, and addressing behavioral science questions. Many review and position papers [6,8-11] are available for the interested reader.

A generally unspoken belief about repeated self-report measures is that repeated assessments conducted throughout a study—regardless of the study duration or the momentary measurement frequency—are thought to be invariant regarding how questions are interpreted, how response scales are construed, and how internal reference standards are applied to ratings. All of these are important considerations for achieving reliable and valid data. That is, we assume that the quality and integrity of responses to all assessments are not affected by repeated measurement processes. However, if this assumption of invariance does not hold, it becomes challenging to disentangle true within-person changes from shifts in measurement quality over time. For example, observed within-person patterns could be erroneously attributed to genuine change when they instead reflect evolving interpretations of questions or changes in response scale use, thereby compromising the validity of inferences about within-person dynamics and processes.

Our primary intention in this viewpoint is to highlight several features of repeated momentary assessments that we think deserve attention, given the possibility that they are associated with changes in momentary data quality. We present selected results to illustrate the point. Because the findings presented later have been reported previously, it is reasonable to assume that they may be present in at least some repeated momentary studies and, importantly, *may* systematically impact data quality and warrant further research. In the same spirit, we do not argue that the features presented are ubiquitous in EMA studies.

We now describe how intensive longitudinal momentary measurements may be distorted by repeatedly asking the same or similar questions over many repetitions. Theoretically, these distortions may be particularly germane when assessments occur many times a day, with only hours between assessments [12], but they may also occur at longer intervals. There is already substantial evidence that responses are not invariant over time, given that repeated measures can create practice effects, a phenomenon supported by abundant research on memory and performance [13-15]. Practice effects could be salutary, leading to faster and more accurate responses [16], or pernicious, leading to unwanted measurement effects such as increased bias and error [17]. In either case, the key point is that repeated exposure to the content can change how questions are answered. To date, evidence on the *adverse* consequences of repeated assessments as they pertain to EMA has been available in a piecemeal fashion spanning several content domains, making it difficult to fully appreciate the scope and importance of the issue.

## Response Times Decrease for EMA Question Completion Over Repeated Measures

### Overview

Although perhaps not well known to EMA researchers, studies examining the time taken to complete an EMA assessment have reported decreases in response time (time it takes individuals to complete questions) over repeated measures, consistent with the practice effects mentioned previously [18-20]. Response time reductions are likely due to respondents’ increasing familiarity with the task, with the wording and meaning of questions, and with the response options over repeated assessments [21]. These decrements can be striking, with reductions of more than 50%. Aggregated response times usually follow a negative exponential pattern, with the steepest decrements occurring early during repeated measures.

**Figure 1:**
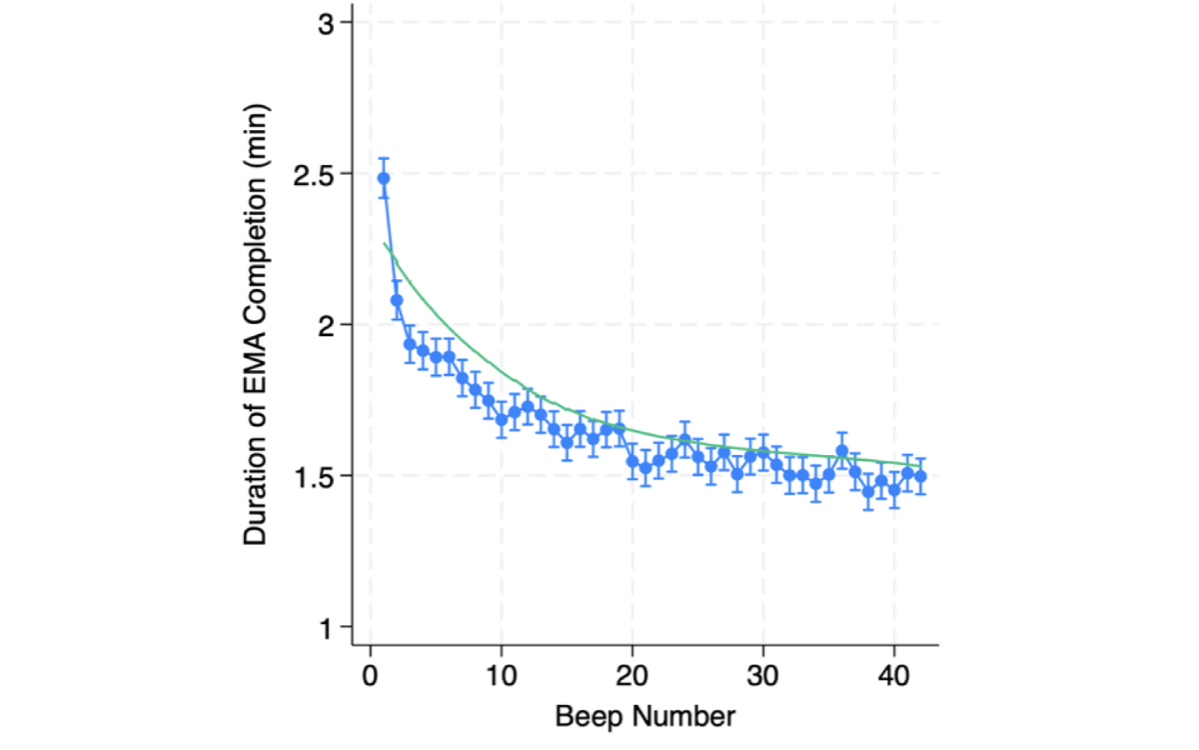
Response times over prompts. EMA: ecological momentary assessment.

To illustrate this pattern of response times over time in an EMA study, data from the Understanding American Study conducted at University of Southern California are shown in Figure 1. A total of 22,531 prompts were recorded from 706 community-dwelling adults, and up to 42 prompts were completed per week. Average response time is plotted for each prompt, revealing a dramatic drop across the first 9 prompts followed by a modest decline over the remaining prompts. Over the course of a week, there was a 32% decline in response times. The magnitude of the decrease in survey completion time is influenced by multiple factors, including the burden of the interview, incentives for completion, and the composition of the participant sample.

### Implications

We conclude that the time to complete assessments may decrease over repeated measures. We speculate that if such changes are associated with, or caused by, any of the factors discussed later, data quality may change over repeated measures. For example, increasing missingness and carelessness over time may generate faster response times and, consequently, compromise data quality. Given this possibility, we suggest that researchers examine the response time changes over the course of repeated measures, though we also acknowledge that response time may or may not be associated with validity.

## Rate of Data Missingness May Increase Over Repeated Measures

### Overview

One plausible explanation for faster survey completion with repeated measures is that individuals gradually disengage from the study procedures and, therefore, spend less time completing assessments. At the start of a study, participants may be motivated to complete all questions and generate optimal answers but may lose interest and become less invested as they repeatedly answer the same questions [22]. When this happens, participants may show increasing noncompliance with the study protocol and may miss assessments when prompted, a concern that has previously been acknowledged by EMA researchers [23].

To assess the magnitude of change in missed EMA prompts over time, we examined many EMA studies with a wide range of samples and variety of assessment schedules. Some studies showed a drop in compliance rates over time, across both shorter and longer studies, and with various frequencies of EMA prompting. Therefore, we suggest that repeated measures can induce *some level* of missingness in EMA studies.

We now turn to the question of whether changes in missing data are serious contenders for inducing bias and how such missingness may affect data quality. If shifts in missingness over time are minimal or if compliance rates remain high overall despite an increase in missed assessments, there is likely little cause of concern. However, in the presence of substantial increases in missingness, missing data can introduce a range of problems, including reduced statistical power, unrepresentative momentary samples (or changes in sample representation), and potential distortions of longitudinal effects. Increasing rates of missing values imply that missing values do not occur completely at random, and researchers may wish to routinely incorporate study day (or prompt number) as an auxiliary variable or covariate in statistical analyses to reduce potential bias due to systematic missingness patterns (ie, to increase the plausibility of the assumption that data are missing at random. Although statistical methods (eg, multilevel multiple imputation [24]) can help account for missing-at-random mechanisms, it is not possible to fully protect against potential patterns that are missing not at random, and only a few studies have developed or evaluated missing data methods specifically for intensive longitudinal contexts, such as EMA data [25].

### Implications

Repeated measures may create bias when missingness increases to substantial levels over the course of a study, and we suggest that missingness over time be considered in intensive repeated-measures studies. Unfortunately, the precise proportion of missingness that would signal caution depends on the cause of this missingness (eg, simple burden or a change in activities that encourage missingness, such as more exercise). Our recommendation is to implement as many safeguards as possible to encourage the most complete data throughout an entire study. This could entail a variety of methodological maneuvers: creating EMA designs that reduce respondent burden related to questionnaire length, daily sampling frequency and scheduling, and study duration; incentive structures that encourage high compliance (eg, payment being contingent on prespecified completion rules from the investigators); and implementing procedures for monitoring compliance over time and providing appropriate feedback to encourage compliance (eg, real-time or daily signaling to the research team about missed prompts). Further research would be welcome to determine which of these strategies are most effective, so that efforts to reduce missingness could be implemented efficiently.

## Frequency of Careless Responding Over Time

### Overview

Careless responding (or insufficient effort responding) is defined as respondents providing answers without regard to the content of the questions, and it can occur when they do not read an item or do not pay attention to what the item is asking [26,27]. Careless responding can also occur as a more subtle form of participant disengagement compared with the overt noncompliance discussed previously, in which a respondent misses prompts entirely. To reduce the burden associated with repeated assessments, respondents may minimize the effort expended by doing the least possible to satisfy study requirements [22]. Careless responding can present as invariable responses (eg, *straight-lining*, in which the same answer is given to every question) or as inconsistent or random responses. A recent small study interviewed individuals at the close of a many-month EMA study to better understand the burden and other issues that could affect data quality [28]. The authors found that respondents tried to counter the burden by responding quickly and that repeated measures over time led to more neutral responses.

Many studies have shown the detrimental effects that careless responding can have on measurement accuracy and reliability [26]. Careless responding can occur when respondents are asked to complete long questionnaires. For instance, toward the end of lengthy questionnaires with dozens or hundreds of items, respondents often give more random, uniform, and fast responses, suggesting that their motivation has waned over time [29,30]. An intuitive extrapolation of these findings is that the repetitive nature of completing brief surveys may similarly lead to more careless responding. Ganzach and Bulmash [31] documented less variable and less complex patterns of self-reporting over the course of daily repeated measures on affect and stress. These findings demonstrated that increased carelessness could occur over repeated measures [32]. Whether decreases in survey completion time can be attributed to increases in extremely fast responses (an indicator of carelessness) has also been shown to occur. In another EMA study, the frequency of speed responding increased from 3% to 8% across 49 assessments over 7 days [33]. Again, we are not saying that all studies, or even most studies, show this pattern—only that it can occur in repeated-measures studies. Another method for assessing changes in carelessness over time is by comparing self-report ratings with objective assessments of the same concept. With increased carelessness, associations over repeated measures would be predicted to decrease because of the increased measurement error. One study found a decreasing association between self-reported and objectively measured time spent on social media in adolescents, which could have been generated through carelessness [34].

### Implications

Available evidence shows instances of increasing carelessness over densely repeated measures. We recommend future work to examine possible changes in careless responding over time. The addition of attention check questions might also improve engagement and reduce carelessness [28]. A fruitful direction for future research could be the development of advanced statistical models, including machine learning techniques, to detect careless responding in datasets in which EMA self-reports can be compared with objective measures. Furthermore, the longitudinal aspect of intensive repeated-measures data may prove a fertile ground for extending carelessness detection approaches, for instance, by examining shifts in within-person parameters (eg, variability, autocorrelation, and outliers) over time.

## Reactivity to Question Content May Increase Over Repeated Measures

### Overview

Reactivity, or reactive arrangements, has been recognized since the 1930s, when workers at a manufacturing facility in Hawthorne, New Jersey, altered their behavior in response to their actions being observed by scientists [35], even though an alternative interpretation has been offered [36]. Here, we restrict our inquiry to reactivity associated with repeated measures in intensive momentary designs, namely, the repeated answering of the same or similar questions many times. This section is divided into 3 subparts: reactivity producing a change in a targeted behavior or cognition, reactivity producing a change in the reporting of a behavior or cognition (but without corresponding change in the targeted phenomenon), and specific reactivity processes associated with changes in question meaning or scale recalibration. These are not exclusive designations, and it is possible that studies exhibit blends of these types of reactivity.

### Reactivity in Behavior, Cognition, and Emotion

This type of reactivity is defined as a change in actual behavior, cognition, and emotion over time due to repeated responses. In fact, some interventions have intentionally incorporated this type of reactivity to induce desired outcomes, such as the use of self-monitoring to affect physical activity behaviors [37,38]. Despite its potential utility in intervention contexts, this phenomenon poses a threat to the internal validity of an observational study as it signifies that measurement is not invariant over time. For example, repeated assessments may cause participants to become more conscious of their behaviors, which may, in turn, trigger self-regulatory mechanisms [39]. The same process may happen with behaviors and emotions. Unfortunately, very few studies are available on whether and how this phenomenon unfolds in EMA studies over time (eg, potential for cumulative effects) [40].

### Reactivity in Reporting

A second type of reactivity is defined by respondents altering the way they *report* behaviors, cognitions, and emotions, independent of whether those behaviors or states have actually changed over time (the previous type of reactivity). In repeated-measures studies, this phenomenon could manifest as a shift in the validity of the reported variables over repeated assessments. For example, reports of affect might be accurate at the onset of a study but gradually shift to become inaccurate or less reliable later; the converse might also be true, as discussed later [18]. A problem for reporting reactivity is that most studies cannot distinguish between real change and reporting change because doing so requires objective measurement of the variable of interest, which is very uncommon in the constructs being measured in EMA. This requirement presents a challenge for the assessment of internal states, where changes over time may indicate actual change, reporting change, or a combination of both.

### Reactivity Processes

The third section on reactivity discusses several ways that actual levels of, or reports of, behavior, cognition, and emotion might change over time through specific psychological processes that may be inherent in repeated measures.

Initial elevation is defined as higher levels of a variable when measured early in a study compared with levels observed at later measurements when there is no apparent reason for a shift in levels over time [41-43]. Although recognized for many years, a paper by Shrout et al [43] has renewed interest in the phenomenon. They found an initial elevation effect for momentary negative states and for momentary internal states in 4 studies with repeated measures, with small to medium effect sizes. Furthermore, Anvari et al [44] (2023) studied several thousand college students and reported a strong initial elevation bias. Thus, initial elevation has been observed, although there is some competing evidence [45] consistently. Biasing of overall levels of variables can result from such effects, for example, by producing (apparently) incorrect downward trajectories in variables over time.

Another facet of reactivity that has not received much attention is that the *meaning of questions* may evolve over repeated administrations of questions [46]. Following up on previous work by Windle [47] (1994), Knowles et al [48] (1996) explicitly explored the possibility that the interpretation of questions changes over time. Results indicated that over multiple exposures to items, individuals gained knowledge about the construct being studied (an anxiety questionnaire). Moreover, they observed shifts in question meaning within a single test administration. Later interpretations of anxiety items demonstrated that a higher standard for endorsing anxiety was evident; if all else remained constant, this would result in diminished reported anxiety later in the study. The upshot of such shifts in question meaning over repeated measures over time is clear: what we learn at the beginning of a study might not be comparable with what we learn later.

Another aspect of question meaning is *response* *scale recalibration*, defined as a shift in how participants use question response options that occurs as a reaction to events experienced throughout the study [46]. Exposures to events that elicit very intense emotions or other strong reactions (eg, pain of childbirth or trauma) may alter the way individuals interpret a rating scale. Such recalibration could result in a stimulus that was once rated at a certain intensity to later receive a lower intensity rating because the upper end of the scale was redefined by the extreme emotion or reaction. Alternatively, recalibration could be induced in certain treatment modalities intended to reduce reported symptomatology through cognitive and social manipulations [49]. Therefore, recalibration could have a major impact on interpreting results over time.

### Implications

It is fundamental to know if repeatedly measuring a variable creates any of the reactivity processes, given that they would be a threat to internal validity. If there is a high likelihood for reactivity, we recommend preliminary investigations to assess the potential magnitude of these effects and to incorporate design strategies to mitigate them, while acknowledging the extra financial and investigator burden inherent in this recommendation. More broadly, if empirical evidence suggests that reactivity is likely (eg, respondents having access to feedback that has been previously shown to induce reactivity), steps could be taken to eliminate the reactivity-inducing aspect of the protocol.

An existing barrier to the application of this approach is that we simply do not have an adequate way to identify reactivity-inducing circumstances. Of particular importance is the possibility that processes and events with the potential of shifting means or recalibrating scales are not routinely measured in longitudinal studies; we therefore recommend routine collection of such data to signal possible distortion. In addition, experimental manipulations of item administration (eg, item order) have been recommended as useful methods to detect possible biases from measurement reactivity [19]. Advancing methodologies to statistically control for these shifts is also recommended; however, some efforts to accomplish this have not resulted in reduced bias, such as using the *then-test* to control for shifts from pretest to posttest [49].

## Conclusions

The aim of this viewpoint was to consider the possibility that studies using intensive repeated self-report measures could distort interpretations of data collected over time. We found evidence supporting the potential for all factors considered: response times can be considerably reduced, missing data rates can increase, carelessness rates can rise, and reactive effects (including reference standards and question meaning) may manifest over repeated measures. Response time reductions alone may or may not impact data quality, as practice effects could be positive or negative. However, when reduced response times lead to increases in missingness, carelessness, and reactivity, there is the potential for compromised data quality. Heightened awareness of these possibilities is warranted for researchers using repeated assessments. We also offered suggestions for monitoring and reducing these possible effects.

We view this discussion as a supplement to prior papers that suggested *guidelines* for conducting, reporting, and analyzing EMA studies. Those papers have guidelines that could be helpful for examining some threats to validity across repeated measures, although not with an eye toward invariance over time. As early as 2002, an EMA guidelines publication [50] indicated the need for comprehensive reporting of procedures and enumerated ways of calculating prompt compliance, which are useful for understanding the threats to validity discussed here. The early set of guidelines did not mention tracking the duration of prompt completion or methods for assessing careless responding. A guidance document [51] included the recommendation to record prompt duration; these data would be pertinent for evaluating the issues raised in this paper. However, their suggestions did not mention tracking careless responding. Overall, these recommendations are positive developments for the field that should result in more robust EMA studies.

In summary, we believe a cautious position is warranted regarding the possibility that repeated questionnaire measurements affect how questions are answered. It is plausible that some results from repeated-measures studies have been affected by the processes discussed previously. This paper calls for research directed at understanding whether reactivity distorts data collected through intensive repeated measures. A speculative extension of this conclusion concerns repeated measures taken at longer intervals, such as every week or month: such longitudinal data may also be susceptible to the biases reported here, with potentially far-reaching implications.

## Abbreviations

EMA: ecological momentary assessment
mHealth: mobile health
